# *Helicobacter pylori* Eradication Efficacy of Therapy Based on the Antimicrobial Susceptibility in Children with Gastritis and Peptic Ulcer in Mekong Delta, Vietnam

**DOI:** 10.3390/children9071019

**Published:** 2022-07-08

**Authors:** Loan T. T. Le, Tuan A. Nguyen, Nghia A. Nguyen, Yen T. H. Nguyen, Hai T. B. Nguyen, Liem T. Nguyen, Mai T. Vi, Thang Nguyen

**Affiliations:** 1 Department of Pediatrics, Can Tho University of Medicine and Pharmacy, Can Tho City 900000, Vietnam; 2 Department of Pediatrics, University of Medicine and Pharmacy at Ho Chi Minh City, Ho Chi Minh City 700000, Vietnam; nguyenanhtuan@ump.edu.vn (T.A.N.); nghianguyen@ump.edu.vn (N.A.N.); 3 Department of Microbiology, Can Tho University of Medicine and Pharmacy, Can Tho City 900000, Vietnam; nthyen@ctump.edu.vn (Y.T.H.N.); ntbhai@ctump.edu.vn (H.T.B.N.); 4 Faculty of Nursing and Medical Technology, Can Tho University of Medicine and Pharmacy, Can Tho City 900000, Vietnam; ntliem@ctump.edu.vn; 5 Department of Pharmacology and Clinical Pharmacy, Can Tho University of Medicine and Pharmacy, Can Tho City 900000, Vietnam; maivivi127@gmail.com

**Keywords:** eradication therapy, *Helicobacter pylori*, children, Vietnam

## Abstract

Background: The efficacy of *Helicobacter pylori* (*H. pylori*) eradication therapy for children is currently low, and antibiotic resistance is a significant cause of treatment failure. The purpose of this study was to evaluate the *H. pylori* eradication efficacy of therapy based on antimicrobial susceptibility in pediatric patients with gastritis and peptic ulcer. Methods: This study was conducted at Can Tho Children’s Hospital and Can Tho University of Medicine and Pharmacy Hospital between March 2019 and April 2022. We performed an upper gastrointestinal endoscopy, cultured *H. pylori* from biopsies of gastric mucosa, determined antibiotic sensitivities to *H. pylori* by the E-test method, and treated eradication based on the antibiotic susceptibilities of bacteria. After at least 4 weeks of eradication therapy, we assessed the effectiveness of treatment with a breath test. Results: Among 237 children recruited in this study, 48.9% were boys and 51.1% were girls, and the mean age was 10.03 ± 2.53 years. We determined that 80.6% of *H. pylori* were resistant to clarithromycin, as well as amoxicillin, metronidazole, levofloxacin, and tetracycline, at 71.7%, 49.4%, 45.1%, and 11.4%, respectively. The overall eradication rate of *H. pylori* was 83.1% (172/207). Among therapies tailored to antimicrobial susceptibility, the bismuth quadruple regimen achieved the greatest success, but the efficacy of triple therapy with esomeprazole + AMX + CLR/MTZ was low. Conclusions: Tailored eradication therapy was highly successful in our study but did not achieve over 90%. We recommend that in countries with a high prevalence of antibiotic resistance in *H. pylori* strains, particularly where the amoxicillin-resistance rate of *H. pylori* is high, therapy tailored to antimicrobial susceptibility should be used as first-line therapy, and bismuth and tetracycline should be added to enhance the eradication efficacy in children.

## 1. Introduction

*Helicobacter pylori* (*H. pylori*) infection is usually acquired in childhood, causing chronic active gastritis. Most infected individuals show no clinical symptoms; however, a small number of *H. pylori* infection cases cause serious gastroduodenal diseases, most importantly peptic ulcer disease, stomach cancer, and mucosa-associated lymphoid tissue (MALT) lymphoma [1,2]. Moreover, Vietnam has the highest stomach cancer frequency in southeast Asia [3]. *H. pylori* eradication treatment was not as complicated and was effective many years ago. However, *H. pylori* is presently becoming more difficult to treat worldwide. The effectiveness of the standard three-drug regimen for eradicating *H. pylori* is generally low and considerably varies from country to country. According to the currently recommended therapies of ESPGHAN/NASPGHAN-2017, the initial *H. pylori* eradication therapy should be based on antibiotic sensitivity, and cure rates should be ≥90% to prevent the development of secondary resistant strains of bacteria and the spread of these resistant strains in the community. Further benefits mentioned are the reduction in costs and risks of salvage therapy and, ultimately, the prevention of and reduction in gastric cancer incidence [4,5]. Several researchers worldwide have documented that antibiotic-susceptibility-based eradication of *H. pylori* in children is highly effective [6,7,8]. Furthermore, recent reports on the antibiotic resistance of *H. pylori* bacteria in Vietnam showed that a very high percentage of *H. pylori* is resistant to commonly used antibiotics, and the recommended eradication treatment is necessary. Hence, we conducted this study to evaluate the efficacy of *H. pylori* eradication in pediatric patients with gastritis and peptic ulcer to understand the efficacy of regimens and related factors contributing to accelerating the success of eradication treatment in children.

## 2. Materials and Methods

### 2.1. Setting and Study Design

This study was carried out at Can Tho Children’s Hospital and Can Tho University of Medicine and Pharmacy Hospital from March 2019 to April 2022. The sample size included 237 pediatric patients aged 5 to 15 years who presented with gastrointestinal symptoms, had indications for esophagogastroduodenoscopy (EGD), and had a positive *H. pylori* culture test. The exclusion criteria included pediatric patients who had a history of gastric bypass surgery, used antibiotics or bismuth within 4 weeks before the endoscopy, used PPI within 2 weeks before the endoscopy, or who had a history of being allergic to one of the drugs in the study therapy. Moreover, during the treatment process, pediatric patients were also excluded if they encountered side effects of the drugs in the study regimen and could not continue treatment, failed to adhere to the 14-day eradication regimen, or did not return for a follow-up visit and check breathing at the end of the course of treatment.

EGD was performed after well-trained endoscopists completely anesthetized the patient at Endoscopy Center, the University of Medicine and Pharmacy Hospital and Gastrointestinal Endoscopy unit by the gastroenterology faculty at Can Tho Children’s Hospital. During the upper gastrointestinal endoscopy, we collected 4 gastric mucosa biopsies. One piece from the antrum and one piece from the body were initially taken for *H. pylori* culture; these biopsies were placed in a transportation medium and immediately transferred to the Department of Microbiology, Can Tho University of Medicine and Pharmacy for culturing. One piece in the antrum and one in the body were used for the urease test (NK Pylori test, Nam Khoa Biotek Co., Ltd., Ho Chi Minh City, Vietnam).

All patients were checked for primary gastrointestinal symptoms by using the Gastrointestinal Symptom Rating Scale (GSRS) and were prescribed PPI drugs within 2 weeks while waiting for *H. pylori* culture results.

### 2.2. Helicobacter pylori Culture and Antimicrobial Susceptibility Testing

Biopsies taken at the gastric antrum and body by gastroduodenoscopy were used for the process of *H. pylori* cultures. Biopsy fragments were added to 500 µL of a transportation medium (20% glycerol, 0.9% NaCl in Milli-Q water). Then, the biopsy fragments were ground in a culture medium (100 µL of brain heart infusion (BHI) solution supplemented with 10% fetal bovine serum (FBS)). The following step involved culturing on an agar plate supplemented with 10% lysed sheep blood (Nam Khoa Biotek Co., Ltd., Ho Chi Minh Cỉty, Vietnam), 1% isoVitale, a skin antibiotic mixture, and amphotericin B. The agar plates were incubated at 37°C in a specific microaerobic atmosphere (mixture of O_2_:CO_2_:N_2_ gas in a ratio of 5:10:85, respectively) for 4–5 days. A single colony in a culture medium for 4–5 days was determined based on colony morphology and the features of *H. pylori,* including Gram-negative S shaped bacterium that were urease-, oxidase-, and catalase-positive.

The minimal inhibitory concentration (MIC) of 5 different antibiotics including amoxicillin (AMX), clarithromycin (CLR), levofloxacin (LEV), tetracycline (TET), and metronidazole (MTZ) were determined by an E-test (BioMerieux, Hanoi, Vietnam). According to the standards of the European Committee on Antimicrobial Susceptibility (EUCAST) 2019, which were used to evaluate the susceptibility, the resistance cutoff values are 0.125 µg/mL for amoxicillin, 0.5 µg/mL for clarithromycin, 1 µg/mL for levofloxacin and tetracycline, and 8 µg/mL for metronidazole [9].

### 2.3. Tailored Therapy

The 14-day regimen was tailored based on the antimicrobial susceptibility test results of the *H. pylori* strain isolated from gastric biopsies of every patient. This therapy consisted of a PPI (esomeprazole) and two antibiotic agents to which the *H. pylori* strain was susceptible. Bismuth-based quadruple therapy was used when pediatric patients infected with *H. pylori* strains were only susceptible to TET or AMX. The dosages of drugs were determined according to the 2017 ESPGHAN/NASPGHAN guidelines [4,10,11].

During the treatment process, doctors advised the patient’s families and patients about the treatment schedule, how to use the drug, and possible side effects, and educated patients to increase adherence to eradication treatment. The doctor checked the patient’s adherence to treatment by making a weekly follow-up appointment and checking the number of medications the patient had taken.

### 2.4. Follow up to Assess the Efficacy of H. pylori Eradication Therapy

The judgment of eradication was performed at least 4 weeks after tailored therapy. Patients were evaluated for gastrointestinal symptoms after therapy by GSRS and the efficacy of *H. pylori* eradication therapy by a urea breath test.

### 2.5. Statistical Analysis

Data were analyzed using the Statistical Package for Social Science (SPSS) version 20.0. Descriptive statistical analysis was used to describe the characteristics of the pediatric patients, such as sex, age, residency, gastric disease, and susceptibility to 0 antibiotics of the strains isolated from the clinical samples. The chi-square test was used to correlate the difference between proportions. Fisher’s exact test was used when more than 20% of the expected counts were less than 5. The *t*-test was used to compare two means. A *p*-value less than 0.05 was accepted as statistically significant.

### 2.6. Ethical Issues

The study was conducted according to the guidelines of the Declaration of Helsinki and approved by the Ethics Committee of Ho Chi Minh University of Medicine and Pharmacy (approval code: No. 273/ĐHYD-HĐĐĐ, approval date: 7/4/2019).

## 3. Results

**Figure 1:**
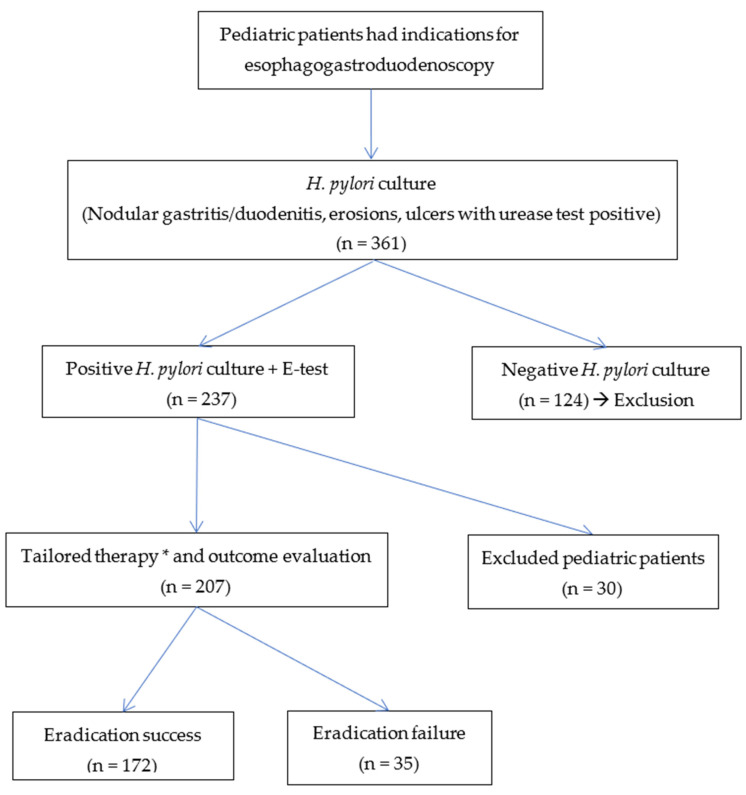
The diagnosis and eradication therapy of *Helicobacter pylori* in the study.

During the study period, we performed a gastroduodenal endoscopy in 750 pediatric patients. Among them, 391 pediatric patients were assigned eradication treatment and underwent *H. pylori* culture. There were 237 pediatric patients with positive *H. pylori* cultures and antibiotic susceptibility testing (Figure 1).

(*) Tailored therapy:

- Susceptibility-guided triple therapy: esomeprazole + amoxicillin (AMX) + clarithromycin (CLR)/metronidazole (MTZ) or levofloxacin (LEV)/adolescent.
- If double resistance:
  + Can only choose one sensitive antibiotic agent: (AMX or MTZ or TET)
    ≤8 years old: bismuth + esomeprazole + high-dose AMX + MTZ.
    Eight years old: Bismuth + Esomeprazole + TET + MTZ.
  + Can choose two sensitive antibiotic agents:
    Esomeprazole + two sensitive antibiotic agents.

### 3.1. Characteristics of Patients

Among the 237 children recruited for this study, 48.9% were boys, and 51.1% were girls. The mean age of patients was 10.03 ± 2.53 years, ranging from 5 to 16; age followed a normal distribution. The highest rate of patients came from Can Tho city (63.3%), and the rest came from the provinces along the Mekong River (Vinh Long, Hau Giang, Soc Trang, Dong Thap, Tien Giang, Ben Tre, An Giang, etc.), accounting for 36.7%. Regarding endoscopic findings, nodular gastritis/duodenitis was the most popular (69.2%), followed by duodenal ulcer (28.7%) and rarely gastric ulcer (2.1%). Almost 1/3 of pediatrics were diagnosed with peptic ulcer (30.8%) and nodular gastritis/duodenitis (69.2%). Approximately 77.2% of strains were isolated from pediatric patients without prior eradication treatment. Among the children who had previously received *H. pylori* eradication therapy, we found that 18/54 (33.3%) patients received a three-drug regimen with CRL, 7/54 (13%) patients received a three-drug regimen with MTZ, and 29/54 patients failed to exploit previous *H. pylori* treatment regimens. Additionally, 90.7% (49/54) of pediatric patients were treated for *H. pylori* once before, and 9.3% of pediatric patients were treated twice (Table 1). Primary gastrointestinal symptoms of the cases are shown in Table 2.

The overall resistance rates to clarithromycin (CLR), amoxicillin (AMX), metronidazole (MTZ), levofloxacin (LEV), and tetracycline (TET) were 80.6% (191/237), 71.7% (170/237), 49.4% (117/237), 45.1% (107/237), and 11.4% (27/237), respectively. None of *H. pylori* strains were sensitive to the five antimicrobial agents. Double resistance with AMX + CLR was 64.2% (148/237), AMX + MTZ was 33.3% (79/237), AMX + LEV was 35% (83/237), CLR + MTZ was 33.8% (80/237), and TET + MTZ was 7.2% (17/237). The resistance to antibiotic agents of *H. pylori* strains is shown in Table 3.

### 3.2. Results of H. pylori Eradication Treatment

Out of the 237 pediatric patients enrolled in the study, 207 were eligible to undergo treatment and evaluate the regimen’s efficacy based on antibiotic sensitivity, and 30 were excluded from the analysis. Among excluded children, 13 patients did not return for the urea breath test, 1 patient was checked by gastroscopy without a breathing test, 1 patient dropped out of treatment due to drug side effects, 7 patients were infected with multi-resistant strains of *H.* *pylori* and their families disagreed with eradication treatment, and 8 patients were not treated according to tailored therapy.

After eradication treatment for at least 4 weeks, 207 pediatric patients were evaluated for clinical symptoms and testing. We found that 192 (92.8%) patients had no clinical symptoms, and 15 (7.2%) patients had mild symptoms; the mean GSRS scores significantly decreased (3.5 ± 1.3/before treatment to 0.09 ± 0.17/after treatment; *p* = 0.00; Cl: 3.23–3.59). The percentage of successful eradication of *H. pylori* was 83.1% (172/207), and treatment failure was 16.9% (35/207) (Table 4).

Regarding the use of *H. pylori* eradication regimens in tailored therapy, 23/207 pediatric patients (11.1%) were treated with EAC, while 32/207 (15.5%) and 3/207 (1.4%) patients were treated with EAM and with EAL, respectively. There were 43/207 (20.8%) patients who used the four-drug regimen with bismuth, and 106/207 (51.2%) of the patients were eradicated with the triple regimen based on antibiotic susceptibility. Regarding the efficacy of regimens, the successful eradication proportion of EAC and EAM regimens in the study was low at 52.2% and 78.1%, respectively. However, the regimen with bismuth and the regimen based on antibiotic sensitivity achieved higher eradication efficacy: 88.4% and 89.6%, respectively (Table 4).

Regarding the factors affecting the effectiveness of eradication treatment, among the 207 treated pediatric patients, the treatment effect in the younger age group (5–8 years old) was 54.5%, significantly lower than that in the older age group (9–16 years old), which was 90% (*p* = 0.00). Similarly, the eradication rate of *H. pylori* in the pediatric group with ulcerative lesions was higher than that in the group with inflammatory lesions (92.1% compared to 79.2%; *p* = 0.02; OR = 0.33, 95% CI 0.12–0.89). There was no statistically significant difference in the efficacy of *H. pylori* eradication treatment by sex, history of previous *H. pylori* treatment, or the use of the regimen with bismuth (*p* > 0.05) (Table 5).

### 3.3. Adverse Effects of Therapy

In terms of adverse effects during *H. pylori* eradication treatment, among the 207 treated children, 29% of the children (60/207) experienced side effects. Most were mild, with no cases requiring hospitalization because of drug side effects. Nausea and vomiting were the most prevalent adverse effect with an incidence of 18.4% (38/207); the metallic test was reported in 8.2% (17/207) of the cases, diarrhea was 7.7% (16/207), anorexia was 3.9% (8/207), and other less common symptoms, such as pain, headache, dizziness, etc., were reported. In addition, there were no complications due to gastroscopy and when performing diagnostic tests for *H. pylori*.

## 4. Discussion

According to the 2017 ESPGHAN/NASPGHAN and 2020 JSPGHAN guidelines, the clinical trial results provided us with necessary information about *H. pylori* eradication based on antibiotic sensitivity in the regions where *H.* *pylori* had a high rate of antibiotic resistance. The eradication rate of *H. pylori* in our study was 83.1% per the protocol, which is lower than the recommendation of ESPGHAN/NASPGHAN of ≥90% [4]. Furthermore, this rate is lower than that reported by Kotilea et al., conducted on 145 children in Belgium from 2011 to 2013, based on antibiotic sensitivity for 10 days, of 89.9% [12]. According to the study by Silva G.M. et al., in Portugal, and Butenko T. et al., in Slovenia, during the period 2013–2017, which considered eradication treatment based on antibiotic sensitivity for 14 days, the percentage of eradication was 97.8% and 85.9%, respectively [7,13]. In Asia, this figure in Ikuse’s study (2016) on Japanese children was 93.8% [6]. According to a pilot study by Zhang Y.D. et al., (2020), which tracked eradication treatment according to antibiotic sensitivity and CYP2C19 phenotype for 14 days, the eradication rate was 99% in 75 Chinese children [8]. Many factors may be associated with the low eradication rate in our study, including mixed infections and host genetic factors. According to the literature, the susceptibility of bacteria to antibiotics in vitro did not always mean successful eradication in vivo [14]. Antibiotic resistance can be acquired during treatment, and a single pediatric patient was likely to be the host of multiple strains of *H. pylori*. Therefore, the eradication rate in this study may be improved if we take multiple biopsies from different stomach sites for cultures, such as the antrum, corpus, and fundus. Additionally, the eradication success rate risked being influenced by host genetics [15]. According to the American Gastroenterological Association, polymorphisms that affected intragastric pH, including those of CYP2C19, IL-1B, and MDR1, are relevant to successful *H. pylori* eradication [16,17].

In Vietnam, research by Tran Duc Long on pediatric patients aged 6–15 years old at Can Tho Children’s Hospital (2017–2019) with peptic ulcer disease, the rate of success of *H. pylori* eradication treatment by a triple regimen (PPIs, amoxicillin, and metronidazole) was only 51.8% [18]. The effectiveness of eradicating *H. pylori* based on antibiotic sensitivity in some recent studies was also relatively low, ranging from 44.7% to 60.8% [19,20,21]. The leading cause of eradication treatment failure is the increasing prevalence of drug-resistant *H. pylori* bacteria in Vietnam. In addition, recent data in Vietnam showed a higher prevalence of amoxicillin resistance than in Europe and North America, ranging from 50% to 71.7% [19,20,21,22]. This threatens the efficacy of eradication in pediatric patients selected for sensitive antibiotics based on standard amoxicillin, metronidazole, and clarithromycin regimens. To overcome the high resistance to amoxicillin, in our study, in the cases of amoxicillin-resistant pediatric patients, we replaced it with tetracycline for children >8 years old or used high-dose AMX and added bismuth for children ≤8 years old. Furthermore, choosing antibiotics that were sensitive to bacteria, we preferred to combine antibiotics that not only had a synergistic effect but were also less susceptible to destruction by gastric acid. The drug stayed in the stomach for as long as possible. Therefore, the eradication efficacy of regimens based on antibiotic sensitivity in our study was significantly higher than in the previous studies.

Regarding the factors affecting the effectiveness of treatment, we found that the bactericidal rate in the younger age group (5–8 years old) was statistically significantly lower than in the older age group (9–16 years old). However, antibiotic resistance did not differ between these two age groups. This may have occurred due to the restriction of the use of some antibiotics by age and the inappropriate use of the drug forms (for example, metronidazole is not available in syrup form). In addition, we found that the eradication rate of *H. pylori* in the group of patients with peptic ulcer lesions was statistically significantly higher than in the group with inflammatory lesions, which was entirely consistent with the literature [23]. Many studies have documented that the eradication rate of *H. pylori* was lower in the group of patients with non-ulcer dyspepsia than in the group with peptic ulcer, which can be explained by the difference in *H. pylori* strains that caused disease in these two groups. The pediatric patients without ulcerative lesions were often infected with *H. pylori* strains with cagA (-), vacA s2, and m2, and had slower growth sequences than those with ulcers. These strains of *H. pylori* will be less susceptible to antibiotic treatment [24]. Interestingly, the efficacy of *H. pylori* eradication based on antibiotic sensitivity was not related to the history of previous *H. pylori* eradication treatments in our study.

The strength of our study is that we studied many pediatric patients aged 5–16 years, using only one PPI, esomeprazole. All pediatric patients were cultured, tested to determine antibiotic sensitivity by the E-test method, and evaluated for effectiveness by a breathing test. These tests are highly specific, allowing accurate evaluation of efficacy. In addition, all pediatric patients were carefully consulted by specialist doctors and adhered well to treatment and follow-up procedures. The weaknesses of our study are the design of an uncontrolled clinical trial, the variability in the study subjects in terms of age and weight, and the lack of proper drug forms that may have affected the drug’s effectiveness. Furthermore, the use of antibiotic drugs in Vietnam is very diverse and challenging to control. Hence, obtaining a complete history of antibiotic use for longer than 1 month is hard before performing gastroduodenal endoscopy, culture, and antibiogram.

Although the eradication rate did not reach >90% as required, the study results provide important information in achieving *H. pylori* eradication in children. Due to the high prevalence of antibiotic resistance in *H. pylori* and the limited number of antibiotics used in the pediatric population, the key to successful initial treatment is antibiotic selection based on the results of the antibiotic susceptibility testing. We considered first-line therapy as double resistance in cases where antibiotic susceptibility testing could not be performed or the test was negative. Additionally, we suggest that further studies should apply molecular biology to individualize eradication treatment, which means considering the influence of host genetic factors (*CYP2C19, MDR1, IL*, etc.) on eradication efficiency, using some new antibiotics or potent stomach acid inhibitors to optimize treatment.

## 5. Conclusions

Due to the increase in antibiotic-resistant strains, successfully eradicating *H. pylori* is challenging. Tailored eradication therapy was highly successful in our study, but it was not over 90%. We recommend that selected eradication regimens be appropriate for the antimicrobial susceptibility in countries with a high prevalence of antibiotic-resistant *H. pylori* strains, especially where amoxicillin-resistance in therapy is high.

## Figures and Tables

**Table 1:** Patient characteristics (*n* = 237).

Demographic Characteristics	Frequency	Rate (%)
Sex		
Male	116	48.9
Female	121	51.1
Age		
Mean age	10.03 ± 2.53	
5–8 years	60	25.3
9–12 years	122	51.5
13–16 years	55	23.2
Geographic area		
Can Tho city	150	63.3
Nearby regions	87	36.7
Endoscopy findings		
Nodular Gastritis/duodenitis	168	69.2
Gastric ulcer	05	2.1
Duodenal ulcer	68	28.7
History eradication		
Without previous therapy	183	77.2
Prior to *H. pylori* treatment	54	22.8

**Table 2:** Primary gastrointestinal symptoms of patients (*n* = 237).

Symptom	Frequency	Rate (%)
Epigastric pain	213	89.9
Periumbilical pain	14	6.1
Pain when hungry	57	24.1
Nausea and vomiting	155	65.4
Heartburn	78	32.9
Constipation	10	4.2
GI bleeding	07	3.0
Anemia	33	13.9

**Table 3:** Antibiotic resistance of *Helicobacter pylori* among pediatric patients (*n* = 237).

Antibiotic Agents	Frequency	Rate (%)
Overall resistance to agents		
CLR	191	80.6
AMX	170	71.7
MTZ	114	49.4
LEV	107	45.1
TET	27	11.4
Mono resistance to agents	24	10.1
Double resistance to agents	78	32.9
Triple resistance to agents	111	46.8
Quadruple resistance to agents	21	8.9
All resistance to agents	03	1.3

CLR, clarithromycin; AMX, amoxicillin; MTZ, metronidazole; LEV, levofloxacin; and TET, tetracycline.

**Table 4:** *Helicobacter pylori* eradication regimens and outcomes.

Regimen	Overall	Eradication Result			
		Success		Failure	
		*n*	%	*n*	%
EAC	23	12	52.2	11	47.8
EAM	32	25	78.1	7	21.9
EAL	3	2	66.6	1	33.4
EB(T/A)M	43	38	88.4	5	11.6
Other triple regimens based on AS	106	95	89.6	11	10.4
Overall eradication rate	207	172	83.1	35	16.9

E, esomeprazole; A, amoxicillin; C, clarithromycin; M, metronidazole; L, levofloxacin; T, tetracycline; B, bismuth; AS, antimicrobial susceptibility.

**Table 5:** *Helicobacter pylori* eradication rate of tailored therapy and associated factors (*n* = 207).

Factor	*n*	Eradication		*p*	OR
		Success	Failure		(95% Confidence Interval)
		*n* (%)	*n* (%)		
Age (years)					
5–8	44	24 (54.5)	20 (45.5)	0.00	8.2 (3.71–18.23)
9–16	163	148 (90.8)	15 (9.2)		
Sex					
Boy	101	84 (83.2)	17 (16.8)	0.98	0.99 (0.4–2.09)
Girl	106	88 (83.0)	18 (17.0)		
Prior treatment					
No	162	135 (83.3)	27 (16.7)	0.86	0.93 (0.39–2.21)
Yes	45	37 (82.2)	8 (17.8)		
Peptic diseases					
Gastritis	144	114 (79.2)	30 (20.8)	0.03	3.05 (1.13–8.28)
Peptic ulcer	63	58 (92.1)	5 (7.9)		
Using bismuth					
Yes	43	38 (88.4)	5 (11.6)	0.3	0.59 (0.21–1.62)
No	164	134 (81.7)	30 (18.3)		

## Data Availability

Not applicable.
